# Silicon-induced thermotolerance in *Solanum lycopersicum* L. via activation of antioxidant system, heat shock proteins, and endogenous phytohormones

**DOI:** 10.1186/s12870-020-02456-7

**Published:** 2020-06-03

**Authors:** Adil Khan, Abdul Latif Khan, Muhammad Imran, Sajjad Asaf, Yoon-Ha Kim, Saqib Bilal, Muhammad Numan, Ahmed Al-Harrasi, Ahmed Al-Rawahi, In-Jung Lee

**Affiliations:** 1 Natural & Medical Sciences Research Center, University of Nizwa, Nizwa, 616 Oman; 2 School of Applied Biosciences, Kyungpook National University, Daegu, 41566 South Korea

**Keywords:** *Solanum lycopersicum* L., Silicon, Heat stress, Antioxidant, Heat shock protein

## Abstract

**Background:**

Abiotic stresses (e.g., heat or limited water and nutrient availability) limit crop production worldwide. With the progression of climate change, the severity and variation of these stresses are expected to increase. Exogenous silicon (Si) has shown beneficial effects on plant growth; however, its role in combating the negative effects of heat stress and their underlying molecular dynamics are not fully understood.

**Results:**

Exogenous Si significantly mitigated the adverse impact of heat stress by improving tomato plant biomass, photosynthetic pigments, and relative water content. Si induced stress tolerance by decreasing the concentrations of superoxide anions and malondialdehyde, as well as mitigating oxidative stress by increasing the gene expression for antioxidant enzymes (peroxidases, catalases, ascorbate peroxidases, superoxide dismutases, and glutathione reductases) under stress conditions. This was attributed to increased Si uptake in the shoots via the upregulation of *low silicon (SlLsi1* and *SlLsi2)* gene expression under heat stress. Interestingly, Si stimulated the expression and transcript accumulation of heat shock proteins by upregulating heat transcription factors (*Hsfs*) such as *SlHsfA1a-b, SlHsfA2-A3,* and *SlHsfA7* in tomato plants under heat stress. On the other hand, defense and stress signaling-related endogenous phytohormones (salicylic acid [SA]/abscisic acid [ABA]) exhibited a decrease in their concentration and biosynthesis following Si application. Additionally, the mRNA and gene expression levels for SA (*SlR1b1, SlPR-P2, SlICS,* and *SlPAL*) and ABA (*SlNCEDI*) were downregulated after exposure to stress conditions.

**Conclusion:**

Si treatment resulted in greater tolerance to abiotic stress conditions, exhibiting higher plant growth dynamics and molecular physiology by regulating the antioxidant defense system, SA/ABA signaling, and *Hsfs* during heat stress.

## Background

Abiotic stress (heat, drought, salinity, and heavy metals) can reduce crop productivity by up to 51–82% [1]. Notably, heat stress is recognized as a major adverse condition affecting yield loss, which is particularly relevant given the current context of global warming and climate change, with temperatures expected to increase by 2 °C [2]. It is predicted that a global temperature increase of 3–4 °C would cause a 15–35% reduction in crop productivity [3]. This scenario will subject crops to a larger range of environmental stressors that could occur simultaneously, leading to severe adverse impacts on crop productivity and future global food security [4]. Physiologically, plants have a well-developed heat stress response (HSR), involving numerous signaling pathways in response to heat stress to minimize or prevent damage during short- and long-term heat exposure [5]. When plants experience heat stress, they undergo morpho-physiological, biochemical, phytohormonal, and transcriptional changes such as osmotic imbalance, enzyme inactivation, reactive oxygen species (ROS) overproduction, and organelle damage, which can lead to cell death [2, 6, 7]. With the predicted increase in the frequency and intensity of heat stress, the rate osmotic potential is significantly hindered by the creation of a cell water potential imbalance, causing tissue damages and influencing essential biochemical pathways. To survive under varying temperature conditions, plants have evolved multiple internal tolerance strategies, such as the secretion of heat shock proteins (HSPs), changes in phytohormone levels, and the scavenging of ROS by different oxidation-reduction enzymes [5]. Furthermore, as molecular chaperones, HSPs play a crucial role in facilitating native protein folding and preventing the irreversible aggregation of denatured proteins. In peanuts, the accumulation of small HSPs was shown to improve heat stress resistance [8]. Studies have also shown that elevated levels of antioxidants are associated with increased thermotolerance [2, 9]. Over-emphasis on individual phytohormones or the interplay of phytohormonal regulation, such as abscisic acid (ABA), salicylic acid (SA), and jasmonic acid play a pivotal role in extending tolerance to heat and drought stress [10, 11]. For example, ABA levels increase significantly following exposure to higher temperatures in peas [12]. To counter the adverse effects of stress, the recruitment of essential metabolites and hormones can be compromised to cope with longer stress duration. Therefore, improving crop stress tolerance is deemed an important undertaking in developing eco-friendly agricultural approaches.

Silicon (Si) is the second most abundant element in the earth’s crust and has frequently been reported to be beneficial for plant growth and development [13, 14]. Due to its strong affinity for other ions, Si is commonly found as silicic acid (H_4_SiO_4_), silicate (xM_12_OySiO_2_), and silica (SiO_2_) [15]. Si can increase plant tolerance to different abiotic and biotic stresses [16–18], such as salt and drought stress [19, 20], extreme temperature stress [21], nutrient deficiency [22], aluminum toxicity [23–26], disease resistance [22, 27], and pest resistance (e.g., from damage caused by wild rabbits, [28]. During stress, Si stimulates multiple response pathways, thereby activating antioxidants, enhancing mineral uptake and organic acid anions, exuding phenolic compounds, and regulating hormonal production [29–33].

Despite the known benefits of Si for plant growth under stress conditions, its underlying mechanisms in alleviating heat stress have rarely been investigated. To the best of our knowledge, only a few studies discussed the heat stress tolerance mechanisms induced by Si application. This study aimed to understand the mechanisms underlying heat stress tolerance to elucidate whether (i) Si extends stress aversion by maintaining active plant growth, (ii) it triggers the production and expression of mRNA transcripts related to antioxidative stress responses, (iii) it activates HSR by activating signaling cascades of HSP-related transcription factors and (iv) it influences stress-responsive ABA and SA syntheses. We also hypothesized that heat stress induces a reduction of plant growth and metabolism by overproducing ROS, stress-hormones, and osmotic pressure, which in turn can be minimized by exogenous Si application.

## Methods

### Plant growth treatment and conditions

Tomato seeds (*Solanum lycopersicum* L.) were obtained from Wadi Al-Lawami International, Oman that did not require any identification procedures and then thoroughly washed and soaked in autoclaved distilled water for 72 h. Uniformly germinated seeds were transplanted into seed trays containing peat moss (moisture content 38.5%, pH 4.5–5.5, electrical conductivity 2.0 dS m^− 1^, bulk density 0.7–1.0 mg m^− 3^, grain size 125–250 μm, with 91.1% organic matter: N, 800–2500 mg kg^− 1^; P, 150–850 mg kg^− 1^; Na, 340 mg kg^− 1^; NaCl, 850 mg kg^− 1^). Seedlings were irrigated with distilled water for 3 weeks to reach three internodes. Tomato seedlings of equal height and leaf number were then transplanted into larger pots (10 × 9 cm) with 200 g of peat moss. Distilled water was applied for another week to acclimatize the plants before the start of the experiment. Four-week-old (third-internode) tomato seedlings were subjected to a completely randomized design with three replicates, each consisting of 15 seedlings subjected to exogenous Si treatment. Two conditions were tested: (i) normal growth and (ii) heat stress with (+Si) and without Si (−Si; only DW). To +Si plants, 50 mL of 1 mM Si was applied in the form of sodium metasilicate (Na_2_SiO_3_), adjusted to pH 7 using HCl. Previous experiments on soybean, tomato, and rice showed that using a 1 Mm Si concentration was most beneficial. After 15 days of Si application, plants were subjected to heat stress (43 ± 0.5 °C). For heat stress, growth chamber conditions were adjusted to (12 h light − 30 °C with 6 h heat − 43 ± 0.5 °C; and 12 h dark − 30 °C) and 60% relative humidity. To mimic normal growth conditions, the growth chamber was set to a 12:12-h dark: light cycle at 30 °C and 60% relative humidity). To gradually increase the temperature, hence changing the growth chamber conditions from those optimized for normal growth to a high-temperature cycle, a 60 min timer was used to increase the temperature gradually by 5 ± 0.5 °C from 30 °C to 43 ± 0.5 °C. Once heat stress conditions were achieved, Si was applied to the +Si group of tomato seedlings using the foliar method (10 mL daily), while –Si seedlings were treated with DW (10 mL daily). Both groups of seedlings were then left under heat stress conditions for 10 days, after which plant growth attributes (shoot length, stem diameter, and the number of leaves) were recorded and the shoot and aerial parts of the plant were harvested and stored in liquid nitrogen until further analysis.

### Chlorophyll pigments and leaf relative water content (LRWC)

Photosynthetic pigments were extracted from 200 mg of tomato seedling leaves and mixed with 80% acetone. Chlorophyll *a* (Chl *a*) and *b* (Chl *b*) contents were estimated according to a previously described method [34]. The absorbance of Chl *a*, Chl *b* and carotenoids was recorded at 663, 645, and 480 nm respectively. Chlorophyll content was calculated using the following equations:

$$ \mathrm{Chl}\ a\ \left(\mathrm{mg}/\mathrm{g}\ \mathrm{FW}\right)=\left[\left\{\left(12.7\times \mathrm{A}663\right)-\left(2.69\times \mathrm{A}645\right)\right\}/1000/\mathrm{W}\right]\times \mathrm{V} $$

$$ \mathrm{Chl}\ b\ \left(\mathrm{mg}/\mathrm{g}\ \mathrm{FW}\right)=\left[\left\{\left(22.9\times \mathrm{A}645\right)-\left(4.68\times \mathrm{A}663\right)\right\}/1000\times \mathrm{W}\right]\times \mathrm{V} $$

Where A is the absorbance and FW is the fresh weight of the leaf sample.

The LRWC was measured according to the method described by Cao, et al. [35]. Second leaves were excised and their fresh mass (FM) was also determined. After being left to float on deionized water for 5 h, saturated mass (SM) was recorded. Leaves were then dried at 80 °C to a constant weight and the dry mass (DM) was measured. The LRWC was calculated using the following equation:

$$ \mathrm{LRWC}\ \left(\%\right)=\left[\left(\mathrm{FM}-\mathrm{DM}\right)/\left(\mathrm{SM}-\mathrm{DM}\right)\right]\times 100 $$

### Silicon analysis by inductively coupled plasma mass spectrometry (ICP-MS)

Si was quantified with 0.05 g of ground samples of freeze-dried tomato roots and leaves according to the method described by Bilal, et al. [36] using inductively coupled plasma mass spectrometry (ICP-MS; Optima 7900DV, Perkin-Elmer, United States).

### RNA extraction and quantitative real-time PCR (qRT-PCR)

The RNA extraction buffer (0.25 M NaCl, 0.05 M Tris–HCl, pH 7.5, 20 mM, EDTA, 1% w/v sodium dodecyl sulfate [SDS], 4% w/v polyvinyl pyrrolidone) was prepared using the protocol described by Liu, et al. [37]. Before adding the sample, 750 μL of the extraction buffer and chloroform: isoamyl alcohol (CI; 24:1 v/v) were placed in a 2-mL RNase-free microcentrifuge tube, then β-mercaptoethanol (40 μL) was added. Next, samples were carefully transferred to the buffer before thawing. The mixture was incubated at 20 °C for 5–8 min followed by centrifugation 12,000×*g* for 10 min at 4 °C. Approximately 600 μL of supernatant was transferred to a 2-mL tube and the same volume of CI was added to all samples. The solutions were mixed gently and centrifuged at 12000×*g* for 10 min at 4 °C. The upper layer was carefully transferred to a 1.5-mL microcentrifuge tube and 1/10 volume of 3 M sodium acetate (pH = 5.2) was added. An equal volume of absolute ethanol was added and the samples were incubated for 30 min at 4 °C. After incubation, samples were centrifuged again at 12000×*g* for 10 min at 4 °C and RNA was recovered. The pellet was dissolved in 200 μL of diethyl pyrocarbonate (DEPC)-treated water and 500 μL of 10 M LiCl was added to the solution. The solutions were then mixed gently and placed on ice for 60 min. Finally, samples were centrifuged once more at 12000×*g* for 10 min at 4 °C and the pellet was washed with 70% ethanol. After removing the ethanol, the pellet was air-dried, then dissolved in 50 μL DEPC-treated water. The quality of the RNA was assessed via agarose gel electrophoresis and quantified using a Qubit RNA broad range kit.

The extracted RNA (> 100 ng/μL) was used for cDNA synthesis. High-Capacity cDNA Reverse Transcription Kit from Thermofisher was used for cDNA synthesis. Master Mix was prepared using 10X RT Buffer, 25X dNTPs Mix, MultiScribe™ Reverse Transcriptase, 10X RT random primers, and nuclease-free water. RNA was added to Master Mix following the desired concentration (e.g., for each 100 ng/μL RNA, 10 μL was taken for cDNA synthesis). PCR was performed in a thermocycler under specific conditions (25 °C for 10 min, 37 °C for 2 h and 85 °C for 5 min). The synthesized cDNA was refrigerated at − 80 °C until further analysis.

The synthesized cDNA was used for gene amplification (Table 1). In total, 19 genes related to HSR, Si transport, drought tolerance, antioxidant enzymes, and the SA and ABA biosynthesis pathways were identified in each sample. *Actin* gene was used as a reference. Forward and reverse primers of 10 pM were used for all genes. For each sample, triplicate reactions were performed to minimize errors and contamination. The reaction was performed under the following conditions: 94 °C for 10 min, 35 cycles of PCR reaction at 94 °C for 45 s, 65 °C for 45 s, 72 °C for 1 min. Finally, the extension temperature was set at 72 °C for another 10 min. A threshold of 0.1 was set for gene amplification. Data were obtained in triplicates and was calculated using CT values and housekeeping gene fold expression patterns reported in Schmittgen and Livak [38].

**Table 1:** List of the primer used for gene expression by qRT-PCR

ID	Forward	Reversed	Encoding protein	Reference	Accession Number
CAT	GTCGATTGGTGTTGAACAGG	AGGACGACAAGGATCAAACC	Catalase	doi:10.4236/ajps.2010.11004	M93719.1
APX	GACTCTTGGAGCCCATTAGG	AGGGTGAAAGGGAACATCAG	cytosolic ascorbate peroxidase	doi:10.4236/ajps.2010.11004	DQ099420.1
POD	TTAGGGAGCAGTTTCCCACT	AGGGTGAAAGGGAACATCAG	peroxidase	doi:10.4236/ajps.2010.11004	DQ099421.1
GR	TTGGTGGAACGTGTGTTCTT	TCTCATTCACTTCCCATCCA	glutathione reductase	doi:10.4236/ajps.2010.11004	AW033378
Cu/Zn-SOD	GGCCAATCTTTGACCCTTTA	AGTCCAGGAGCAAGTCCAGT	SOD	doi:10.3390/molecules23030535	Solyc11g066390
GST	TACTCGTTTTTGGGCTCGTT	CACCGATTCAACTCCCTCTG	GST	doi:10.1371/journal.pone.0054880	olyc01g086680
GPX	ACGGAGCAAGCGACAATTGACAAC	CGATTGATTCACCGCAAAGCTCGT	GPX	doi:10.1371/journal.pone.0054880	Solyc08g080940
NCED1	CTTATTTGGCTATCGCTGAACC	CCTCCAACTTCAAACTCATTGC	Synthesis of abscisic acid	Nitsch et al. (2009)	Z97215
ICS	TGCTGCCTCATGGACATACC	TGCGAATGGGGATTTTTCTT	isochorismate synthase	Not reported	XM_019214147.2
PAL	CACTTGTGAATGGCACAGCA	TCCGTTCATCACTTCAGCAAA	phenylalanine ammonia-lyase 1	Not reported	XM_004234584.3
PR1b1	GCACTAAACCTAAAGAAAAATGGG	AAGTTGGCATCCCAAGACATA	Signal pathway of salicylic acid	Tucci et al. (2011)	Y08804
PR-P2	GGAACAGGAACACAAGAAACAGTGA	CCCAATCCATTAGTGTCCAATCG	Signal pathway of salicylic acid	Tucci et al. (2011)	X58548
HsfA1a	GGGATAAATGAGGCAGCAAA	TTGACCTGCAATTGCTGAAG	HsfA1a	doi: 10.1104/pp.15.01913.	Solyc08g005170
HsfA2	CTCACCCCATTCAGGTGTTT	TGCTGCAATGGACAATGAAT	HsfA2	10.1104/pp.15.01913	LOC101255223
SlHsfA3	AGATCCCTTGCAGGTAGCTG	TGATGGCAGTATCCCAATGG	Heat Stress Transcription Factor	doi:10.1371/journal.pone.0054880	
HsfA7	GCTTCTTTTATCCATGGTGTCC	CTTGAACCTGGAAACTCTTC	Heat Stress Transcription Factor	doi:10.1371/journal.pone.0054880	Solyc09g065660
HsfA1b	GAAAGCTTGCACTGACGCAGG	GGTCCGATATGATAGATAGTG	Heat Stress Transcription Factor	doi:10.1371/journal.pone.0054880	Solyc03g097120
DREB2	ATGATAATAATGTCTACAGAGCAA	CTAATGTTGCCATAAAAAACTCTC	dehydration responsive element binding	DOI 10.1007/s13580-011-0125-5	
MAPK1	ATGCGCTTACAGAGGAACAGATG	CGGACGGAATGCACACATATATAC	enhanced drought tolerance	10.1007/s11240-017-1358-5	AJ535702

### Determination of oxidative stress during heat stress

The extent of lipid peroxidation based on malondialdehyde (MDA) was determined in a previous study [39]. In our assay, 10 mM phosphate buffer (pH 7) was used to prepare the tissue homogenate. The reaction mixture consisted of 1.5 mL of 20% acetic acid (pH 3.5), 0.2 mL of 8.1% SDS, 1.5 mL of 0.81% thiobarbituric acid (TBA), and 0.2 mL of tissue homogenate. The reaction tube was heated for 60 min. The reaction mixture was then placed at room temperature for 15 min, followed by the addition of 5 mL of the butanol: pyridine (15:1 v/v) solution. The upper organic layer (i.e., the pink solution) was collected and the optical density was recorded at 532 nm. Tetramethoxypropane was used as an external standard and the experiments were performed in triplicates.

The generation rate of O_2_^−^ was measured using the method described in Gajewska and Skłodowska [40]. Fresh plant powder (1 g) was immersed in phosphate buffer (pH 7) containing 10 mM sodium phosphate, 0.05% (w/v) nitrobluetetrazolium (NBT), and 10 mM sodium azide (NaN_3_). The mixture was kept for 1 h at room temperature, then, 5 mL of the solution was transferred into a new test tube and heated for 15 min at 85 °C in a water bath. The solution was then cooled on ice and vacuum filtered. The absorbance of the sample was read at 580 nm with a spectrophotometer. The experiment was performed in triplicates.

### Quantification of antioxidant enzymes

For the quantification of total protein, a protein extract was prepared by grinding 100 mg of leaf sample with potassium phosphate buffer (100 mM; pH 6.8) containing 0.2 mM EDTA. After centrifugation for 30 min at 12,000×*g*, the supernatant was transferred to a new tube for the determination of total protein content. The protocol described by Bradford [41] was used to quantify total protein content. The assay was conducted at 595 nm on a spectrophotometer. The experiment was performed in triplicates.

The protocol established by Kar and Mishra [42] was slightly modified to determine the activity of the antioxidant enzymes peroxidase (POD), catalase (CAT), polyphenol oxidase (PPO), and ascorbate oxidase (APX). To quantify these enzymes, 100 mg of powdered leaf sample was mixed with 0.1 M phosphate buffer (pH 7). The resulting mixture was centrifuged at 10,000 rpm and 4 °C for 30 min in a refrigerated centrifuge. To quantify POD, 100 μL of the crude extract was combined with 0.1 M potassium phosphate buffer (pH 6.8), 50 μL H_2_O_2_ (50 μM), and 50 μL pyrogallol (50 μM). The reaction mixture was incubated at room temperature for 5 min, followed by the addition of H_2_SO_4_ (v/v) (5%). The extent of purpurogallin production was measured based on the optical density at 420 nm. To quantify PPO, we used a similar reaction mixture to that used for POD quantification but added H_2_O_2_ (50 μM), and the final assay was conducted at 420 nm. CAT activity was determined using the method developed by Aebi [43]: the protein mixture was combined with 10 mM phosphate buffer (pH 7) and supplemented with 0.2 M H_2_O_2_. CAT activity was measured by the decrease in absorbance at 240 nm and expressed as μg of H_2_O_2_ released/mg protein/min. To assay APX activity, 100 mg of fresh plant powder was immersed in 1 mL of 50 mM phosphate buffer solution (pH 7) containing 1 mM EDTA and 1 mM ascorbic acid, followed by homogenization at 50 Hz for 30 s. The resulting homogenates were centrifuged at 4830×*g* at 4 °C for 15 min. Subsequently, the supernatant was combined with the phosphate buffer (pH 7) containing 0.3 mM H_2_O_2_ and 15 mM ascorbic acid. The reaction mixture was then analyzed spectroscopically at 290 nm. One unit of APX was defined as the variable quantity of absorbance at 290 nm per minute.

### SA extraction and quantification

SA was extracted and quantified from freeze-dried tomato samples according to the method developed by Seskar, et al. [44] and described by Shahzad, et al. [45]. The extracted samples were subjected to high-performance liquid chromatography (HPLC) performed using a Shimadzu device outfitted with a fluorescence indicator (Shimadzu RF-10AxL) with excitation at 305 nm and emission at 365 nm, filled with a C18 reverse phase HPLC column (HP Hypersil ODS, particle size 5 μm, pore size 120 Å, Waters). The flow rate was maintained at 1 mL/min. The experiment was repeated three times and each time comprised three replications.

### ABA extraction and quantification

Endogenous ABA was extracted and quantified according to the modified protocol described by Shahzad, et al. [46] and Bilal, et al. [36]. Briefly, samples extracted from ground freeze-dried plants were supplemented with [(±)-3,5,5,7,7,7-d6]-ABA as an internal standard and further analyzed using gas chromatography-mass spectroscopy (GCMS; 6890 N network GC system) and a 5973-network mass selective detector (Agilent Technologies, Palo Alto, CA, USA). To expand the affectability of the method, spectra were recorded for the selected ions at m/z 162 and 190 for Me-ABA, and at m/z 166 and 194 for Me-[2H6]-ABA. Moreover, ABA was calculated from the value of the endogenous peak in comparison to the respective standard. The experiment was repeated three times and each time comprised three replications.

### Statistical analysis

All experiments were performed in triplicates and data collected from each repetition were pooled together. All values are presented as the mean ± standard deviation (SD). Means were analyzed using Duncan’s multiple range (DMRT) tests, with significance set at *P* < 0.05. All analyses were conducted using SAS 9.1 software (Cary, NC, USA).

## Results

### Effects of exogenous Si application on tomato plant growth parameters

**Fig. 1:**
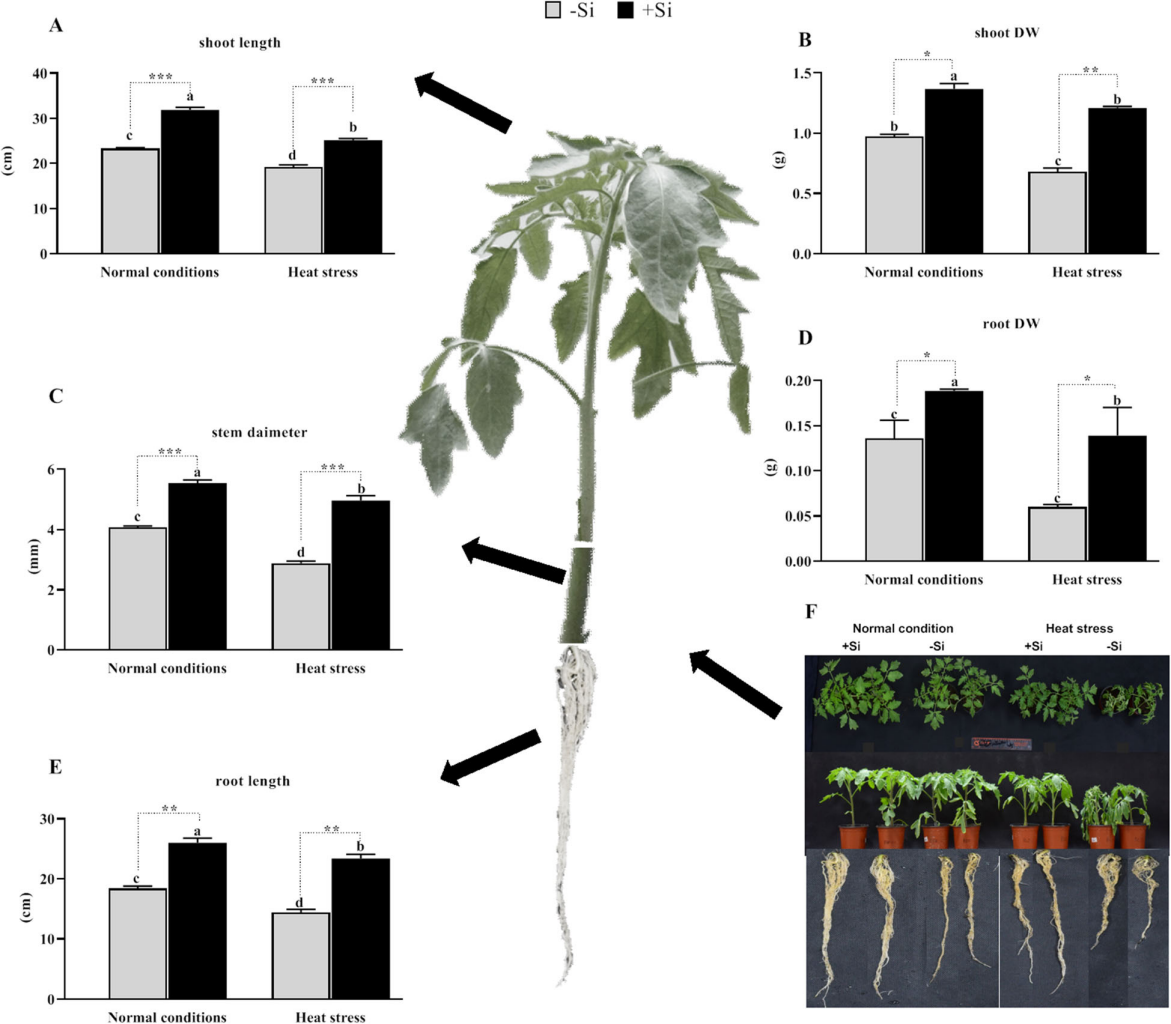
Effects of silicon (Si) application on the growth parameters of tomato plants grown under normal and heat stress conditions. **a** Shoot length, **b** Shoot dry weight, **c** stem diameter, **d** root dry weight, **e** root length, **f** effect of Si on the phenotype of tomato plants in the presence or absence of heat stress. Values are presented as the mean ± SE (*n* = 15). Different letters in one measure indicate a statistically significant difference at *P* < 0.005

In this study, exogenous Si application increased growth parameters under both normal and heat stress conditions. The results showed that exogenous Si application significantly increased shoot length under normal and heat stress conditions compared to –Si (36 and 31%, respectively; *P* < 0.001; Fig. 1a). Pronounced wilting of the leaf was prolific in –Si plants compared to the condition in +Si plants. This was further validated by the significant increase in stem diameter in +Si seedlings under both conditions (36 and 72%, respectively; *P* < 0.001; Fig. 1c). This suggests an ameliorative effect of Si on plant growth under heat stress. Additionally, shoot biomass also significantly increased in +Si plants under normal and heat stress conditions compared to the results for –Si plants (61 and 70%, respectively; *P* < 0.001; Fig. 1b; Supplementary Fig. S1A). The root length, fresh, and dry biomass decreased under heat stress. However, +Si application significantly improved the root morphological traits and increased root length compared to the levels in –Si plants under heat stress and normal conditions (41 and 62%, respectively; *P* < 0.001; Fig. 1e), as shown by the secondary and tertiary root development. Similarly, Si application significantly increased the root and fresh root weights in both normal and heat stress conditions (42 and 28%, respectively, *P* < 0.001; 74 and 62%, respectively, *P* < 0.001; Fig. 1d; Supplementary Fig. S1B).

### Effects of exogenous Si application on chlorophyll pigments and relative water content

**Fig. 2:**
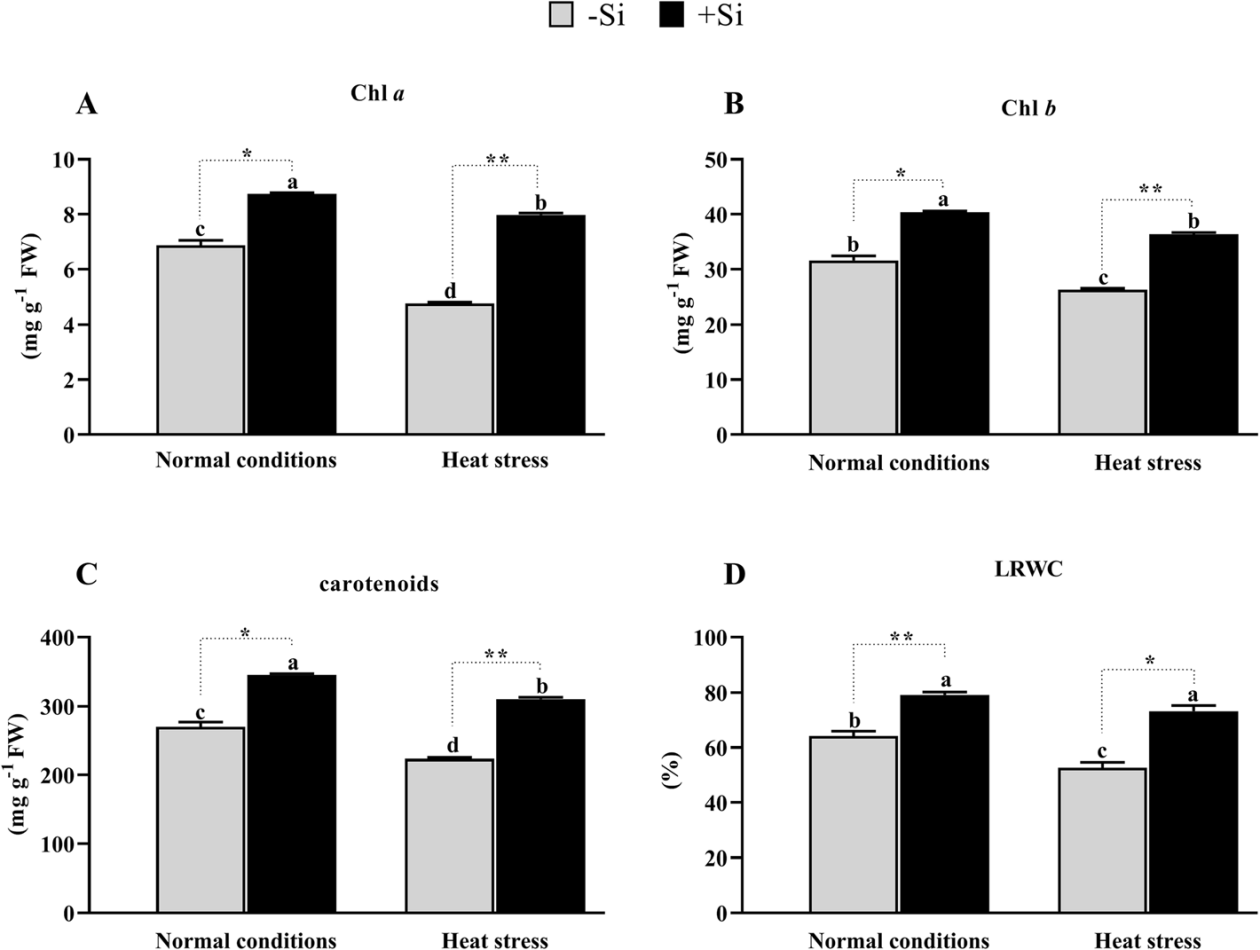
Effects of Si application on photosynthesis. **a** Chlorophyll *a* (**b**) Chlorophyll *b* (**c**) Carotenoids (**d**) leaf relative water content (LRWC) under control and heat stress conditions. Values are presented as the mean ± SE (*n* = 6). Different letters in one measure indicate a statistically significant difference at *P* < 0.005

Si application significantly increased Chl *a* (27 and 38%, *P* < 0.001), Chl *b* (27 and 38%, P < 0.001), and carotenoid (27 and 39%, P < 0.001) content in normal and heat-stressed tomato plants. By contrast, significant reductions in Chl *a*, Chl *b,* and carotenoid content (19, 19, and 21%) were recorded in heat-stressed –Si plants (Fig. 2a-c). This suggests that Si mitigated the adverse effects of heat stress by improving Chl *a*, Chl *b,* and carotenoids. Given the predicted impact of heat stress on plant osmotic potential, we also analyzed LRWC. A significant increase of 13 and 20% (*P* < 0.001) in the water content was recorded in +Si plants compared to –Si plants under normal and heat-stressed conditions, respectively (Fig. 2d). Heat stress increased the plant’s vulnerability to water stress and plants with an adequate supply of Si were more hydrated than –Si plants.

### Effects of Si on superoxide anion (O_2_^−^) and MDA

**Fig. 3:**
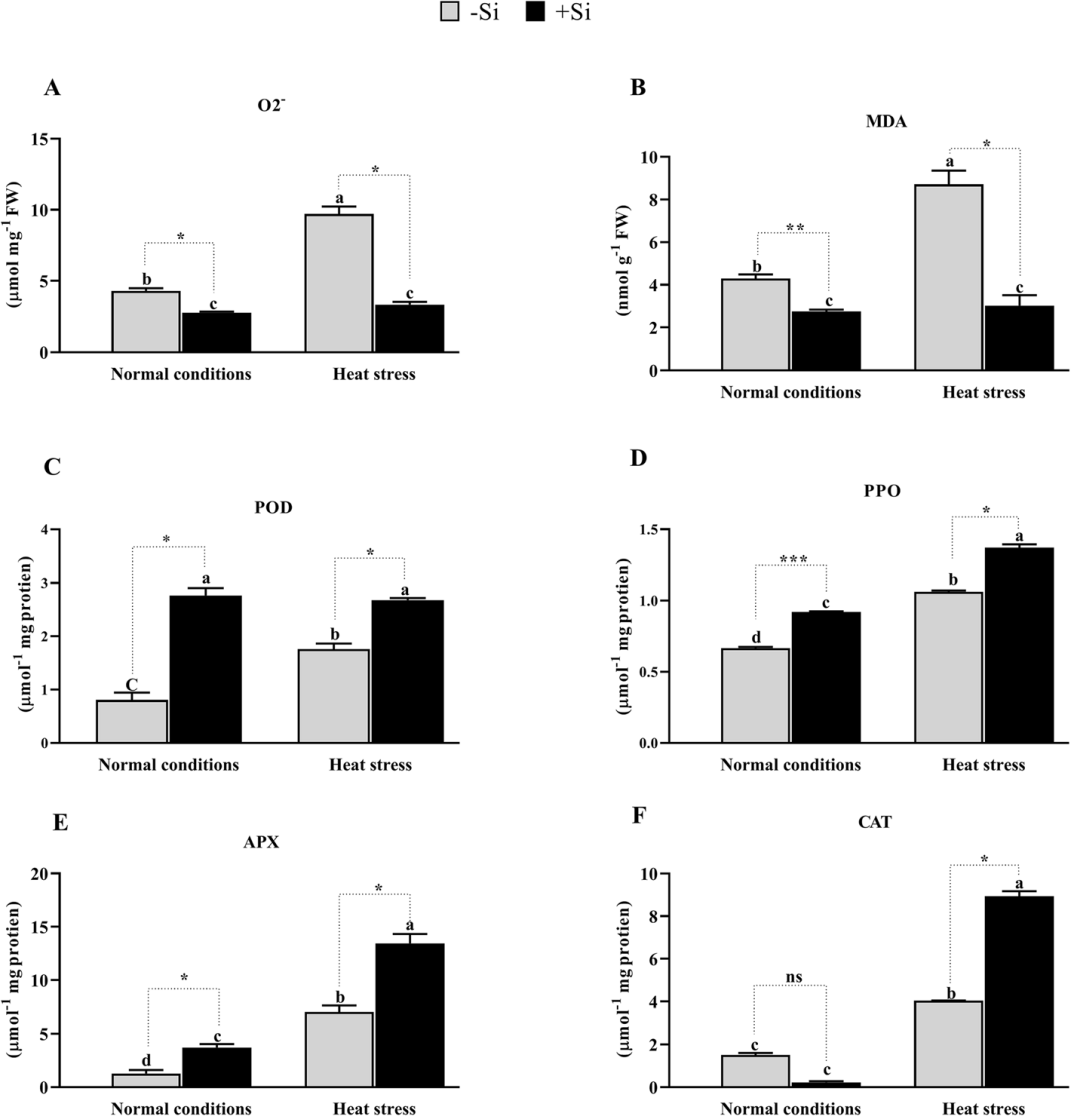
Effects of Si application on stress-related parameters. **a** Malondialdehyde (MDA) content and (**b**) In situ O2−, **c** Peroxidase (POD), **d** Polyphenol oxidase (PPO), **e** ascorbate peroxidase (APX) and (**f**) catalase (CAT). Each data point presents the mean of three replicates. Means denoted by different letters are significantly different (*P* < 0.05)

The ROS-induced peroxidation of lipid membranes is a reflection of stress-induced cell damage [47]. One of the main products of ROS is O_2_^−^, which causes significant damages to cellular organelles and their respective functions [48]. In this study, we found that the production of O_2_^−^ increased significantly by 77.9% (*P* < 0.001) in heat-stressed conditions in –Si plants compared to the production in normal growth conditions (Fig. 3a), while in +Si plants, the production of O_2_^−^ increased by only 30.2% under heat stress. This increase was significantly lower than that in –Si plants, indicating the regulatory role of +Si during heat stress and the generation of ROS.

Furthermore, we quantified the level of MDA under –Si and + Si conditions (Fig. 3b). MDA is a by-product of lipid peroxidation and is an indirect indicator of oxidative stress and damage to the lipid bilayer [49]. MDA significantly decreased by 56.0 and 64.5% (*P* < 0.001) in +Si plants compared to –Si plants under normal and heat-stressed conditions, respectively. This suggests that Si subverted the process of lipid peroxidation compared to –Si during heat stress.

### Antioxidant enzyme synthesis and mRNA gene expression profiling in –Si and + Si plants

Our experiments revealed an increased production of POD, CAT, SOD, and PPO in +Si plants compared to –Si plants in both the normal and heat-stressed conditions. However, under normal growth conditions, CAT activity was lower in –Si plants but increased in +Si plants under heat stress. Under normal growth conditions, POD activity was also significantly higher in +Si plants compared to –Si plants (79.2%, *P* < 0.001); however, POD activity only increased by 51.77% under heat-stressed conditions (Fig. 3c). Furthermore, the activity of POD in –Si and + Si plants under heat stress increased by 35.4 and 14.6%, respectively, compared to POD activity under normal conditions.

Likewise, in +Si tomato seedlings, PPO, APX, and CAT activities increased by 29.24, 91.2, and 121.6% (*P* < 0.001) under heat-stressed conditions compared to –Si plants (Fig. 3d-f). Under normal growth conditions, however, the activity of PPO and APX increased by 39.4 and 189.1% (*P* < 0.001), respectively, but the activity of CAT decreased by 86.5% (*P* < 0.001) in +Si compared to –Si plants. Figure 3d-f show that the activity of PPO, APX, and CAT in –Si and + Si plants under heat stress increased by 60.6, 449.2, and 166.8% (*P* < 0.001) compared to the normal conditions.

**Fig. 4:**
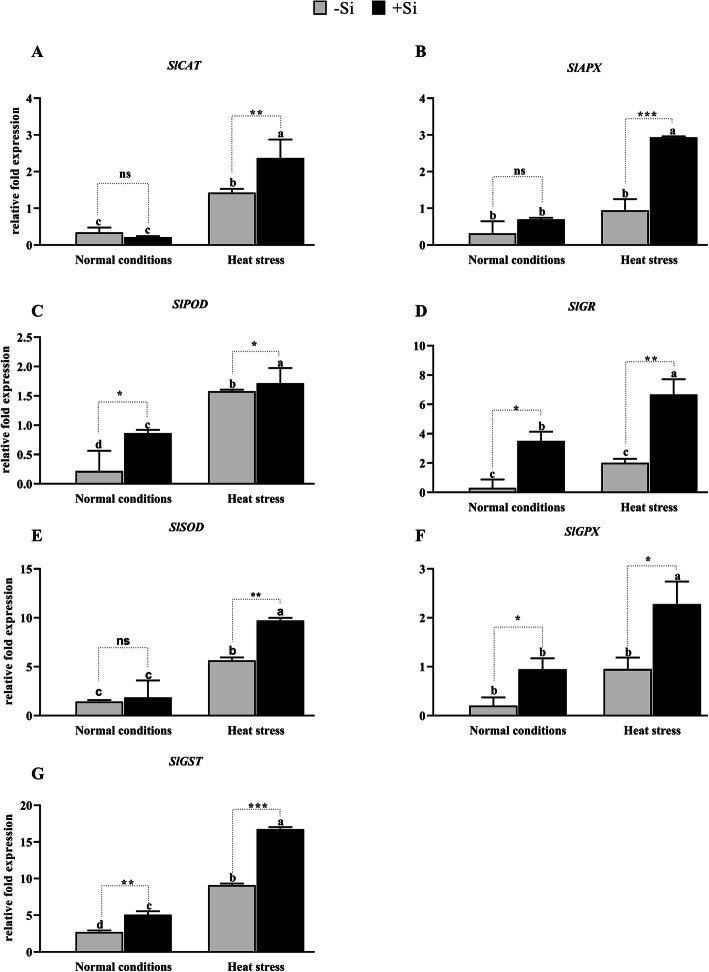
Effect of Si application on the transcript of genes encoding antioxidant enzymes. **a***SlCAT*, **b***SlAPX*, **c***SlPOD*, **d***SLGR*, **e***SlSOD*, **f***SlGPX,* and (**g**) *SlGST*. Each data point presents the mean of three replicates. Means denoted by different letters are significantly different (*P* < 0.05)

To investigate the molecular mechanisms underlying the effects of Si on antioxidant enzymes in tomato plants during heat-stressed conditions, we evaluated the relative mRNA expression levels of the *SlCAT, SlAPX, SlPOD, SlGR, SlSOD, SlGPX,* and *SlGST* genes (Fig. 4). The relative expression levels five of these genes (except *SIGR* and *SIGPX*) were significantly upregulated when –Si tomato plants were exposed to heat stress (*P* < 0.001; Fig. 4a-g). Interestingly, the +Si tomato plants exposed to heat stress showed high relative expression levels of *SlCAT* (2.3-fold), *SlAPX* (2.9-fold), *SlPOD* (1.7-fold), *SlGR* (6.6-fold), *SlSOD* (9.7-fold), *SlGPX* (2.2-fold), and *SlGST* (16.7-fold) compared to that recorded in –Si plants. Under normal growth conditions, the relative expression levels of *SlPOD, SlGR,* and *SlGST* genes increased significantly by 0.86, 3.5, and 5.08-fold (*P* < 0.001) in +Si plants compared to –Si plants, while *SlCAT* was downregulated by 0.21-fold. However, no significant increase was noted in the genes *SlAPX, SlSOD,* and *SlGPX* (0.69, 1.85, and 0.95-fold) respectively (Fig. 4a-g). The increase in the relative gene expression levels in +Si tomato plants compared to those of –Si plants indicates that Si improved the expression of genes involved in antioxidant enzyme production.

### Effects of Si on relative expression level of genes coding for HSFs and stress-related proteins

**Fig. 5:**
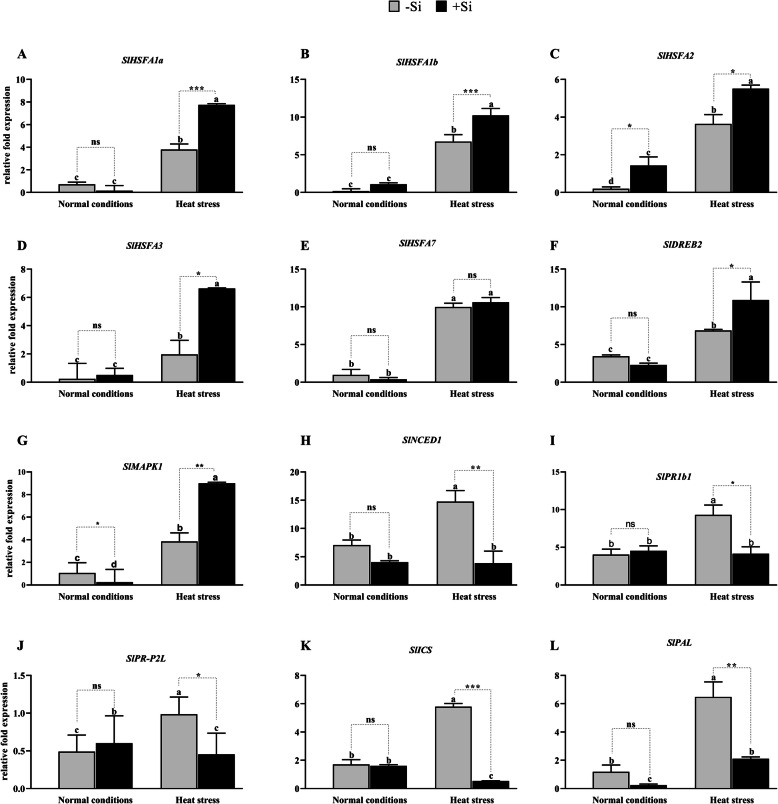
Effect of Si application on the transcript of genes encoding heat shock transcription factors. **a***SlHsfA1a*, **b***SlHsfA1b,***c***SlHsf2A,***d***SlHsfA3,***e***SlHsfA7*, **f***SlDREB2*, **g***SlMAPK*, **h***SlNCED1*, **i***SlPRb1*, **j***SlPR-P2l*, **k***SlICS1,* and (**l**) *SlPAL.* Each data point presents the mean of three replicates. Means denoted by different letters are significantly different (*P* < 0.05)

*Hsfs* regulate the expression of HSPs. In this study, we evaluated the relative mRNA expression level of *SlHsfA1a, SlHsfA1b, SlHsfA2, SlHsfA3,* and *SlHsfA7* under normal and heat-stressed conditions in +Si and –Si plants (Fig. 5a-e). Our results showed that the expression of *Hsfs* and stress-related genes was upregulated by warm temperatures. In +Si plants, a high relative expression level was recorded for *SlHsfA1a* (7.7-fold)*, SlHsfA1b* (10-fold)*, SlHsfA2* (5.5-fold)*, SlHsfA3* (6.6-fold), and *SlHsfA7* (10.62-fold) under heat stress compared to –Si plants. However, under normal growth conditions, there was no significant increase in the relative expression levels of *HsfA1b* and *HsfA3* in +Si plants compared to –Si plants*.* Under normal growth conditions, *HsfA1a* and *HsfA3* were downregulated in +Si plants compared to –Si plants (Fig. 5a-e). Hence, the exogenous Si-mediated increased the expression of *Hsfs* and stress-responsive genes under heat stress. This ultimately triggered the HSR in tomato plants by activating *Hsps,* which consequently led to the prevention of lipid peroxidation and the generation of excessive reactive radicals. Furthermore, the activated HSR further increased the secretion of plant antioxidant enzymes.

The overexpression of transcription factors can modulate a wide range of signaling pathways involved in stress tolerance [50]. The dehydration-responsive element-binding proteins (*SlDREB2)* transcription factors are regulated by abiotic stress-related genes, thus providing the plant with tolerance to various environmental stimuli [51]. The products of these genes are thought to function both in stress tolerance and in the regulation of gene expression and signal transduction of genes related to stress response [52, 53]. Transcription factor *SlDREB2* was weakly expressed in the control plants and was stimulated by heat stress in both –Si and + Si plants by 6.88-fold and 10.91-fold, respectively. These results suggest that Si application increased the expression of *SlDREB2*, thus improving tomato seedlings tolerance to heat stress (Fig. 5f). Previous studies revealed that protein kinases play a similar role as calcium-dependent protein kinases and mitogen-activated protein kinases (*MAPKs*) in response to abiotic stress [54]. In this study, we found that the expression of *SlMAPKs* was induced by heat stress (3.85-fold) in –Si plants, increasing by 9-fold in +Si plants under heat stress (Fig. 5g).

### Effects of Si on endogenous phytohormones during heat stress

**Fig. 6:**
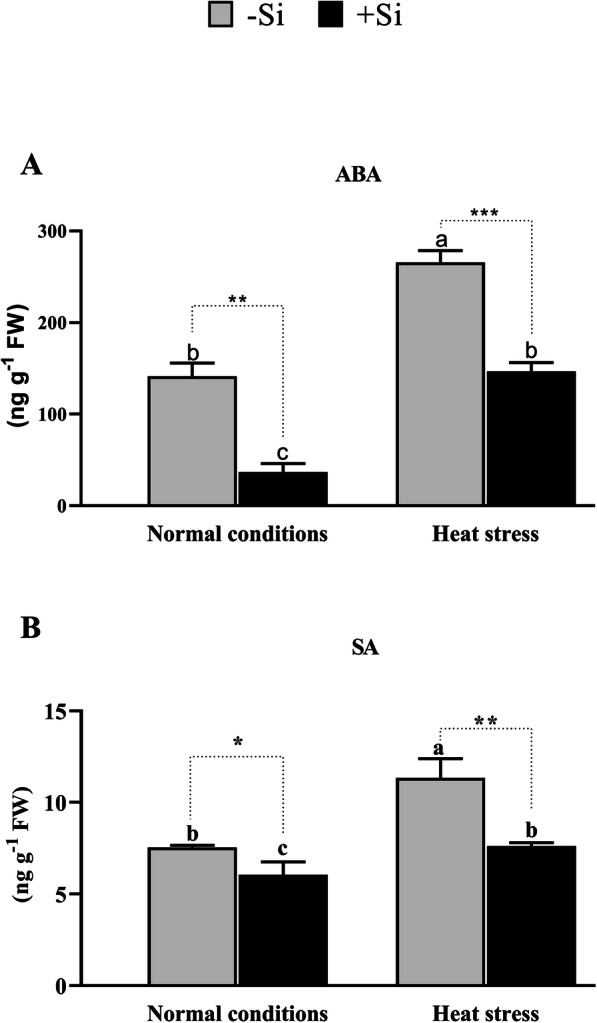
Effect of 1 mM Si application on the level of plant stress tolerance phytohormones (**a**) Abscisic acid (ABA), and (**b**) Salicylic acid (SA). Each data point presents the mean of three replicates. Means denoted by different letters are significantly different (*P* < 0.05)

Plants respond to various abiotic and biotic stressors through alterations in hormonal homeostasis, biosynthesis rate, stability, content, and cell compartmentalization [55]. Phytohormones play an important role in plant growth and development and the current literature suggests these also play a role in the plant’s response to various stresses [56]. In this study, we quantified endogenous ABA and SA hormones in +Si and –Si plants under both normal and heat stress conditions. The results revealed that –Si plants had significantly higher concentrations of ABA (265.91 ng g^− 1^ FW) and SA (11.34 ng g^− 1^ FW) compared to +Si plants under normal growth conditions (141.48 and 7.53 ng g^− 1^ FW, respectively). Furthermore, the application of Si reduced ABA content significantly in tomato seedlings under both the normal and heat stress conditions by 74 and 44%, respectively. Similarly, SA content was reduced by 19 and 32% in plants treated with Si compared to the level in –Si plants under normal and heat stress conditions, respectively (Fig. 6a and b). Our findings indicate that +Si plants produced less ABA and SA, which might be the reason they experienced higher tolerance and a lower amount of heat stress.

The effects of heat stress and exogenous Si on SA pathway-related genes (*SlR1b1, SlPR-P2, SlICS, and SlPAL*) and ABA (*SlNCEDI*) were examined using qRT-PCR (Fig. 5h-l). The change in expression of *SlR1b1, SlPrP2, SlICS,* and *SlPAL* marker genes involved in the SA biosynthesis pathway was strongly and moderately downregulated in the +Si and –Si plants, respectively, under heat stress conditions. Interestingly, these results concur with those observed for the SA levels. However, under normal growth conditions, *SlR1b1* and *SlPR-P2* were slightly upregulated in +Si plants compared to –Si plants, while *SlICS* and *SlPAL* were downregulated, albeit not significantly. The SA signaling pathway appears to be activated under heat stress; however, in the current study, the exogenous application of Si resulted in the downregulation of this pathway. Such results are perfectly consistent with the fact that plant antioxidant capacity is inversely proportional to SA concentration. Furthermore, high concentrations of SA cause cell death or increase vulnerability to abiotic stress. Hence, Si application reduced the biosynthesis of SA, which indirectly enhanced the production of antioxidant enzymes.

### Si, Na, and K concentrations in tomato plants under heat stress conditions

**Fig. 7:**
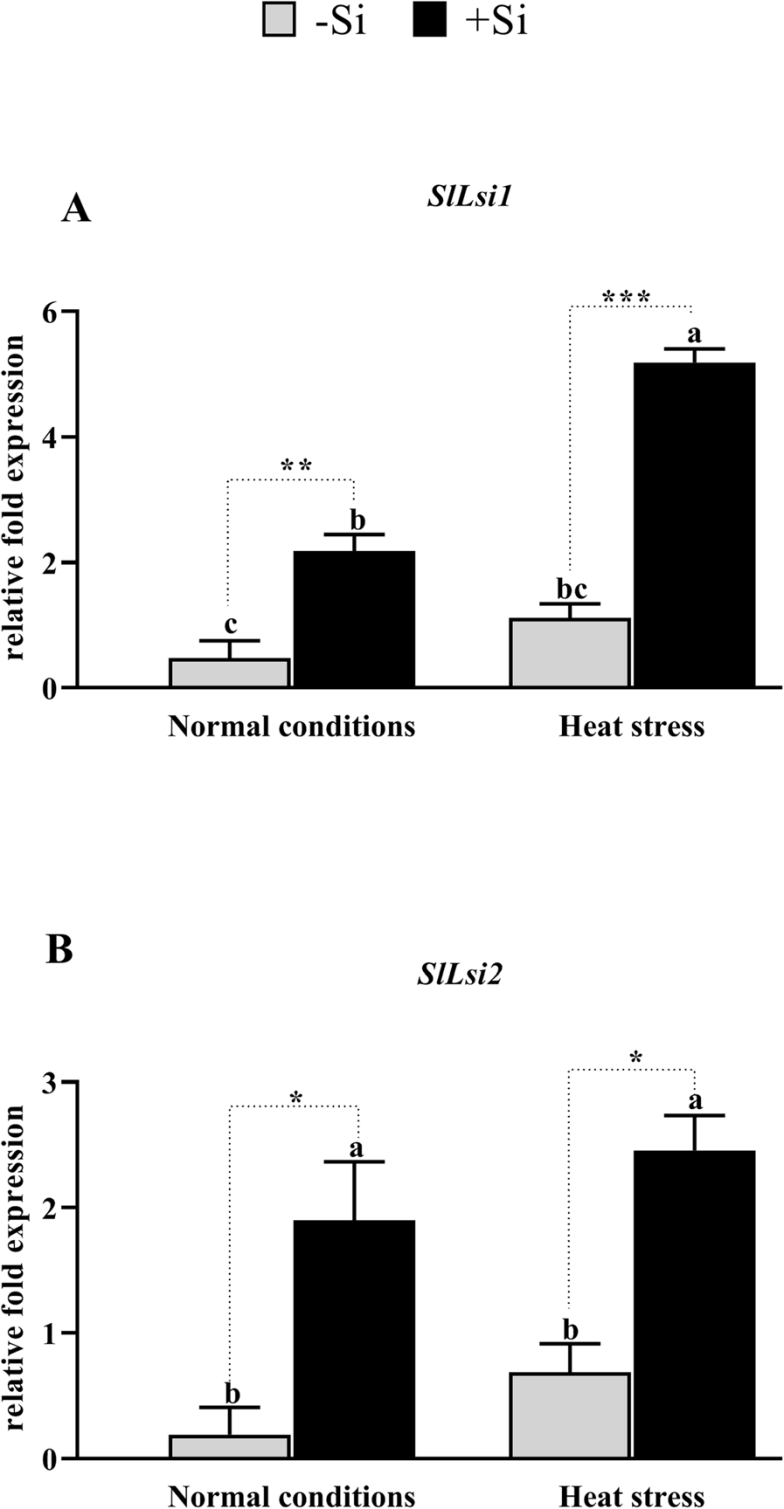
Effect of Si application on the transcript of genes for Si uptake in plants. **a***SlLsi1,***b***SlLsi2*. Each data point presents the mean of three replicates. Means denoted by different letters are significantly different (*P* < 0.05)

Si, Na, and K concentrations were measured in the leaves of +Si and –Si tomato plants after harvest. Unsurprisingly, Si concentration was significantly higher in +Si plants compared to –Si plants in both the normal and heat stress conditions (5.2-fold and 6.1-fold higher, respectively, Table 2). This result was further validated at the gene expression level by investigating the relative expression of the Low Silicon 1 and 2 (*SlLsi1* and *SlLsi2*) genes. As shown in Fig. 7a and b, exogenous Si application improved the expression of *SlLsi1* (2.1 and 5.1-fold) and *SlLsi2* (1.9 and 2.4-fold) under both the normal and heat stress conditions, respectively. On the other hand, Na concentration in the leaves of the tomato plants was not significantly different between +Si and –Si plants. However, the Na concentration was slightly higher in the +Si plants. This result indicates that exogenous Na application in the form of silicate did not increase Na concentration in the leaves of the tomato plants. However, Si significantly (*P* < 0.001) improved the uptake of K from the soil by approximately 0.6 to 1-fold (Table 2).

**Table 2:** Concentrations of Si, Na and K in the tomato shoot under normal and heat stress conditions

Treatments	Concentration (μmol g^− 1^)		
	Si	Na	K
Normal condition (+Si)	2772.82 ± 10.3 ^b^	1071.85 ± 17.3^a^	32,703.64 ± 165.3 ^a^
Normal condition (−Si)	563.06 ± 17.9 ^c^	1080.43 ± 55.6 ^b^	25,561.12 ± 122.8 ^c^
Heat stress (+Si)	3693.89 ± 39.3 ^a^	1270.98 ± 99.7^a^	31,995.73 ± 16.4^b^
Heat stress (−Si)	601.96 ± 31.1 ^c^	1210.23 ± 85.1^a^	20,002.39 ± 79.4 ^d^

Mean ± standard error from three replications per treatment. In the column, the same letters indicate No significant difference (*P* > 0.05) by Duncan’s Multiple Range Test (DMRT)

## Discussion

Heat stress severely hinders crop production, causing substantial losses to the economy and endangering global food security [57–60]. Similarly to trends shown in other crops, it also negatively affects the growth and productivity of tomato plants [61–63]. Our findings demonstrate that exogenous Si application can mitigate the adverse effects pf heat stress in tomato plants by improving plant growth attributes, such as shoot length and biomass production. Similar findings were previously reported in *Cucumis sativus* L. [64], *Nephrolepis exaltata* L. [65], and *Oryza sativa* L. [66]. However, little is known about this phenomenon in tomato plants. The results presented in this study concur with those of previous reports by Mahdieh, et al. [67], Abbas, et al. [68], and Chen, et al. [69], where the application of exogenous Si alleviated the negative effects of abiotic stress and restored plant growth. The growth impacts were inferred from the increased levels in the concentration of photosynthetic pigments (Chl *a*, Chl *b,* and carotenoids) in Si + heat-stressed plants, while studies by [70], Wang, et al. [71], and Chalanika De Silva and Asaeda [72] showed that these concentrations were reduced by heat stress. These reductions were associated with the reduced production of ROS and thereby indirectly representative of the plants stress level [72, 73]. Furthermore, heat stress drastically decreased LRWC by inducing physiological water-deficit. On the other hand, Si application increased LRWC, which led to the increase in photosynthetic pigments observed in the current study. A similar finding was reached by previous studies following during heat stress [74], salinity [75], drought stress [76], and osmotic stress [77]. Similarly to other abiotic stresses, heat stress increased ROS production that consequently oxidize membrane lipids [78, 79] as high concentrations of MDA and O_2_^−^ in –Si plants were observed compared to +Si plants. The triggering of both molecules can lead to the disruption of activities of antioxidant enzymes [80]. Our results were similar those observed after Si application on *Hordeum vulgare* L. [80], *Cicer arietinum* L. [80], and *Nephrolepis exaltata* [65].

**Fig. 8:**
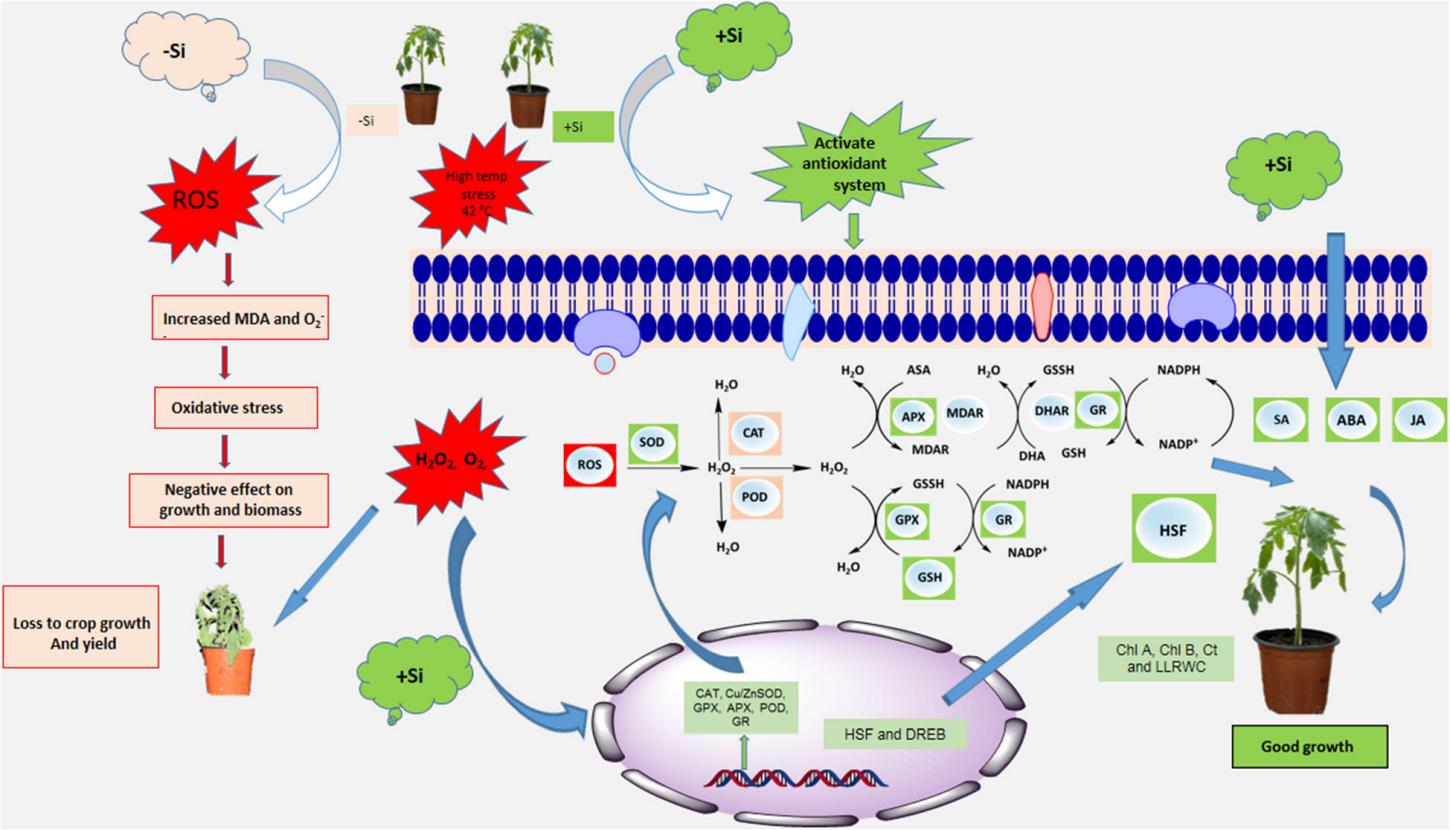
Three strategies are involved in Si enhancement of plant tolerance to high temperatures (43 ± 0.5 °C). In strategy I, Si enhances the antioxidant enzyme activity by both upregulating the expression of antioxidant enzyme genes and reducing the decrease of MDA and O_2_^−^ caused by Reactive Oxygen Species (ROS). In strategy II, Si activates the heat shock proteins and drought tolerance genes such as *SlDREB2* and *SlMAPK1.* In strategy III, Si downregulates stress-related hormones, such as ABA and SA

Plants have evolved an effective antioxidant system using POD, CAT, SOD, PPO, and APX enzymes to eliminate stress-induced excess ROS, thereby protecting the cells from the deleterious effects of oxidation reactions [81]. CAT, APX, and POD antioxidants are known for the dismutation of hydrogen peroxide to water and molecular oxygen in cells [82], as well as the elimination of stress-induced ROS directly or indirectly via the production of ascorbate and glutathione [83]. In this study, we found that the activities of ROS-eliminating enzymes changed significantly. All of the antioxidant enzymes examined in this study, including CAT, POD, PPO, and APX increased significantly in plants exposed to heat stress. However, under normal growth conditions, −Si plants showed higher CAT activity compared to +Si plants. This unusual regulation of CAT activity has been reported in several studies [84, 85]. +Si plants show enhanced CAT activity during heat stress compared to normal conditions (Fig. 8), concurring with previous findings [83, 86, 87]. The roles of Si in genes encoding for antioxidant enzymes have only been studied in a very narrow range. In this study, we elucidated the effects of Si on genes encoding for antioxidant enzymes, such as *SlCAT, SlAPX, SlPOD, SlGR, SlSOD, SlGPX,* and *SlGST* genes. Our findings revealed that Si application in heat-stressed tomato plants increased the relative expression of *SlCAT, SlAPX, SlPOD, SlGR, SlSOD, SlGPX,* and *SlGST* genes, in line with previous reports by Sahebi, et al. [88] and Alberto, et al. [89]. Ma, et al. [90] reported that Si application induces high stress tolerance in plants by upregulating the antioxidant systems of plants.

Heat stress also influences the translocation of essential nutrients [91] and the resulting imbalance can either increase or decrease them. For example, growth inhibition is correlated with excessive Na^+^ concentration and K^+^ deficiency (Fig. 8). Either high Na or low K in the soil are considered as a stress condition that can severely affect plant performance and agricultural productivity (Luan et al., 2009). Give that we used sodium silicate as a Si source in this study, both Si and Na^+^ were analyzed. We did not observe any difference in the Na^+^ concentration in plants treated with and without sodium silicate, suggesting that the observed toxicity was not due to Na^+^ concentration but to high temperatures and induced drought stress. Si uptake was confirmed by measuring the Si concentration in shoots. We also validated the higher uptake of Si in +Si plants at the mRNA level.

The function of *SlLsi1* and *SlLsi2* genes in Si transport and stress conditions are poorly understood. Previously, Ma, et al. [92] described the role of *Oryza sativa OsLsi1* and *OsLsi2*, present in the plasma membrane of rice plant cells, in Si influx and efflux. *OsLsi1* is located on the distal side of the cell while *OsLsi2* is located on its proximal side [92]. Our results revealed that both *SlLsi1* and *SlLsi2* genes were upregulated by 2- and 5-fold and by 1.9- and 2.4-fold in +Si plants under normal and heat stress conditions, respectively. They were also upregulated under heat stress in –Si plants, suggesting they improved Si uptake and played a role in heat stress tolerance (Fig. 8). This role might be attributed to the accumulation of Si in the shoots, providing additional strength to the leaf and stem structure to minimize the adverse effects of heat and drought stress, hence leading to higher tolerance. The role of *OsLsi1* and *OsLsi2* in heavy metal tolerance was previously reported by Kim, et al. [93].

*Hsf*, a regulatory protein, plays a central regulatory role in the conversion of heat stress signal perception to the expression of genes involved in the plant’s stress response by interacting with promoters at the *cis* binding region of HSPs [94]. These in turn activate a cascade of heat stress-responsive genes that work together to improve the plant’s heat stress tolerance. The regulatory role of *Hsfs* has been studied in *Arabidopsis* during heat stress conditions and in other crops [95, 96]. *Hsfs* are conserved in different species, but discrepancies regarding the function of individual *Hsfs* in HSR have also been reported. *HsfA1a* is a master regulator for the initiation of HSR in tomato plants, but in *Arabidopsis*, *HsfA1a, HsfA1b*, and *HsfA1d* are responsible for HSR [97, 98]. Furthemore, under heat stress, *HsfB1* in tomato shows both coactivator and repressor functions, while the same gene in *Arabidopsis* acts as a transcriptional repressor [99]. *HsfA1a, HsfA2*, and *HsfB1* are considered essential for the activation of HSR in tomato plants under heat stress conditions [100] *HsfA1a* and *HsfB1* are produced continuously in the cell, while *HsfA2* is expressed under normal conditions. It was found that the expression of *HsfA2* mRNA and protein increased under heat stress. Consequently, *HsfA2* was more abundant when the plant was grown at higher temperatures [101]. In this study, we found that heat stress significantly enhanced the expression of *Hsfs,* namely *SlHsfA1a, SlHsfA1b, SlHsfA2, SlHsfA3,* and *SlHsfA7*, which varied under normal conditions. This finding highlights the role of *SlHsfA1a, SlHsfA1b, SlHsfA2, SlHsfA3,* and *SlHsfA7* in heat stress tolerance. Interestingly, we found a higher expression of *Hsfs* in +Si plants, leading us to the conclusion that exogenous Si application induced the expression of *Hsfs* and stress-response genes under heat stress. This ultimately activates HSR in tomato plants by activating *Hsps,* leading to the prevention of lipid peroxidation and the generation of excessive reactive radicals. *Hsfs* regulate the transcription changes needed to protect plants from heat stress [8]. Similarly, we found that heat increased the expression of *SlDREB2* and *SlMAPK1.* Wang, et al. [102] previously reported that overexpression of *SlMAPK1* enhanced drought and heat stress tolerance in tomato plants. Moreover, the role of *MAPK1* in abiotic stresses such as cold, salinity, UV radiation, and wounding has also been reported in *Arabidopsis* [103, 104].

Stress-related endogenous hormonal regulation and its related pathways (such as ABA and SA) are considered essential in mediating plant growth and physiological response under stress conditions. ABA signaling and ABA-responsive genes have been extensively studied under a wide range of stress conditions. ABA synthesis triggers ABA-inducible gene expression and causes stomatal closure, thereby reducing water loss via transpiration and eventually restricting cellular growth [105–107]. In this study, significantly higher amounts of ABA were observed in heat-stressed –Si plants compared to +Si plants. This difference indicates that tomato plants experienced high levels of stress. In +Si plants, ABA concentration significantly decreased during heat stress and under normal conditions. These results concur with those Kim, et al. [93], who reported that ABA increased significantly when rice plants were treated with Cu, Cd, and a combination of Cd and Cu in the presence of Si. It has been demonstrated in *Pisum sativum* by Li, et al. [12] that the ABA level increased upon exposure to heat stress, confirming its role in heat stress tolerance. Later, the same group reported that the ABA-deficient mutant tomato genotype is sensitive to heat stress (42 °C for 24 h) [108, 109]. In this study, a significant reduction in ABA was recorded in +Si plants, an indication that +Si plants are less sensitive to heat stress than –Si plants. This was further validated by the gene expression profile, indicating that heat stress upregulated the expression of genes responsible for ABA biosynthesis. We observed higher expression of the *NCED1* gene during heat stress and under normal conditions in –Si plants compared to +Si plants, suggesting that Si application reduced the biosynthesis of ABA.

SA is also a naturally-occurring plant hormone involved in the response to abiotic and biotic stresses and in the regulation of pathogenesis-associated protein expression [110]. The SA response under biotic stress has been widely reported [111]. SA also plays an important role in plant growth, ripening, and development [112] and its role in thermotolerance is well-established. Davies [112] and Li, et al. [113] reported that SA-mediated pathways could increase heat stress tolerance in plant species such as mustard, tobacco, bean, potato, tomato, and *Arabidopsis thaliana*. Similarly, we found that heat stress significantly improved the biosynthesis of SA in our tomato seedlings, suggesting its role in regulating heat stress tolerance. However, the results revealed that exogenous Si application significantly reduced SA concentration. This may be because the plants experienced less stress due to the accumulation of Si in the shoots, forming a protective layer on the leaves. Another possible reason is the interaction with antioxidant systems in the presence of exogenous Si. Li, et al. [113] indicated that high levels of SA caused oxidative stress, while lower levels of SA improved the antioxidative capacity of plants and stimulated the synthesis of protective compounds, leading to enhanced tolerance to abiotic stress. Our results also concur with those of Kim, et al. [93], who reported that exogenous Si application significantly reduced SA biosynthesis in rice plants under heavy metal stress. Under both normal and stress conditions, the synthesis of SA is mediated by two pathways: the phenylalanine ammonia-lyase (PAL) pathway and the isochorismate (IC) pathway. The IC pathway is the major pathway in *Solanum lycopersicum* and *Nicotiana benthamiana* [114]. We found similar results at the mRNA level; SA biosynthesis-related genes (*SlR1b1, SlPR-P2, SlICS, and SlPAL*) were downregulated in +Si plants, but upregulated under heat stress in –Si plants. However, more comprehensive investigations are required to describe the detailed molecular mechanisms underlying the complex roles of SA in abiotic stress tolerance.

## Conclusion

This study showed that Si application to tomato seedlings increased the resilience and function of tomato plants under heat stress and induced stress tolerance by modulating oxidative stress, *HSP,* and endogenous phytohormones, as well as the related mRNA gene expression patterns. Si application reduced the heat-mediated oxidative stress through stimulation of the antioxidant defense mechanism and increased the concentration of photosynthetic pigments in the plant. Thus, using Si for broader field applications with the advent of current changes in global climatic conditions can be an eco-friendly approach to maintain crop growth and productivity.

## Supplementary information

**Additional file 1: Figure S1.** Effects of 1 mM Si application on growth parameters of tomato plants grown under normal and heat stress conditions. (A) shoot fresh weight (B) root fresh weight.

## Data Availability

All the data is available within the manuscript.

## Abbreviations

PAL: Phenylalanine ammonia-lyase
*MAPKs*: Mitogen-activated protein kinases
MDA: Malondialdehyde
*HSP*: Heat shock proteins
SA: Salicylic acid
ABA: Abscisic acids
ROS: Reactive oxygen species
POD: Peroxidase
CAT: Catalase
SOD: Superoxide dismutase
PPO: Polyphenol peroxidase
