# Supplementary figures and images for: Silicon-induced thermotolerance in Solanum lycopersicum L. via activation of antioxidant system, heat shock proteins, and endogenous phytohormones
Source: BMC Plant Biol. 2020 Jun 3;20:248. doi: 10.1186/s12870-020-02456-7 (PMC7268409; doi:10.1186/s12870-020-02456-7)

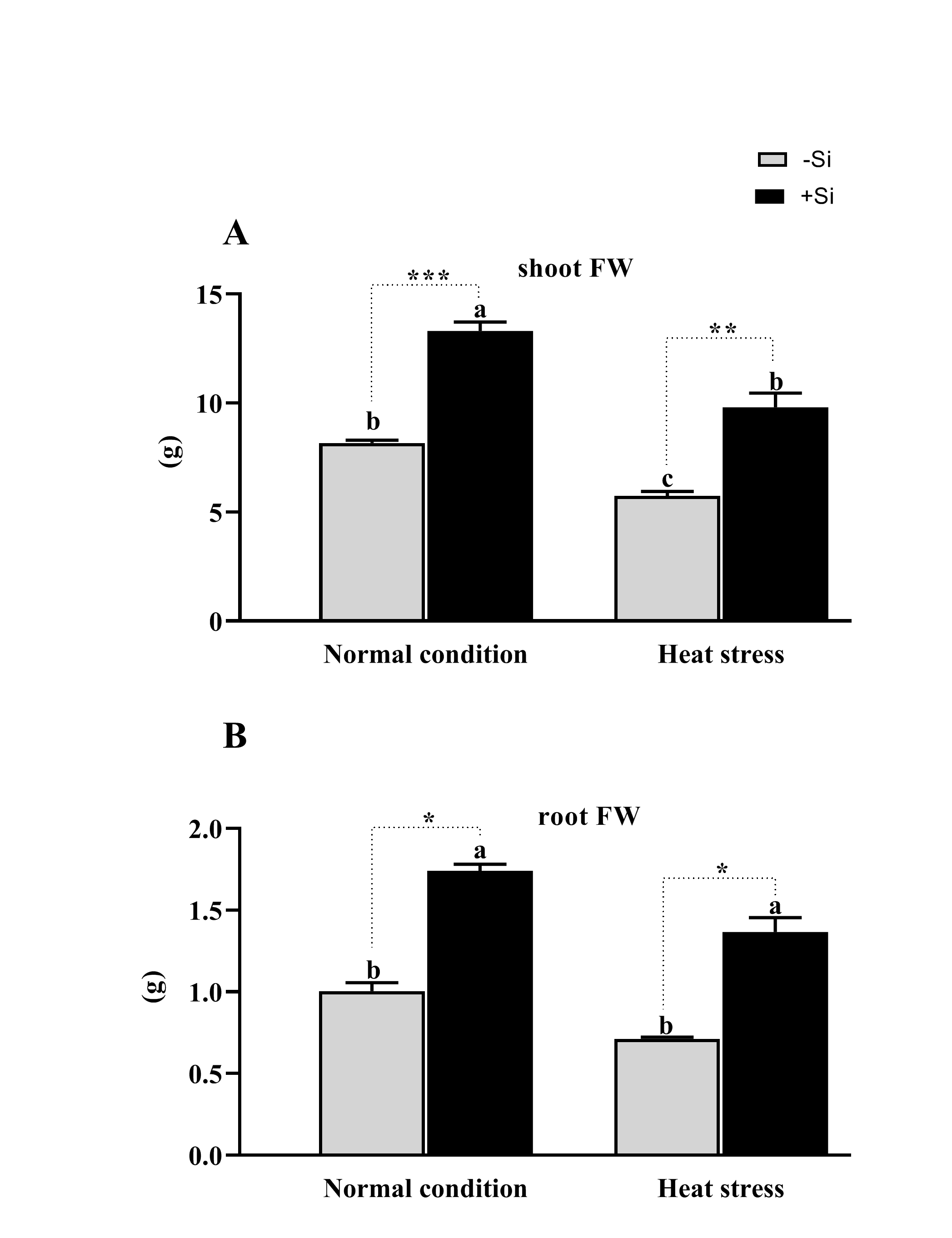

Supplement: Supplementary file 1 — Additional file 1: Figure S1. Effects of 1 mM Si application on growth parameters of tomato plants grown under normal and heat stress conditions. (A) shoot fresh weight (B) root fresh weight. [file 12870_2020_2456_MOESM1_ESM.tif]
